# Cognitive and socio-emotional correlates of psychological well-being and mental health in Guatemalan adults

**DOI:** 10.1186/s40359-021-00654-y

**Published:** 2021-09-23

**Authors:** María J. Ramírez-Luzuriaga, Laura Ochaeta, Manuel Ramírez-Zea, Ann DiGirolamo, Rachel Waford, Charlotte Wray, Reynaldo Martorell, Aryeh D. Stein

**Affiliations:** 1 Nutrition and Health Science Program, Laney Graduate School, Emory University, 1518 Clifton Rd NE, 30322 Atlanta, GA USA; 2 INCAP Research Center for the Prevention of Chronic Diseases (CIIPEC), Institute of Nutrition of Central America and Panama, Guatemala City, Guatemala; 3 Georgia Health Policy Center, Georgia State University, Atlanta, GA USA; 4 Hubert Department of Global Health, Rollins School of Public Health Emory University, Atlanta, GA USA; 5 Department of Psychiatry, Medical Sciences Division, University of Oxford, Oxford, UK

**Keywords:** Psychological well-being, Executive function, Spirituality and religion, General intelligence, Mental health, Social support, Structural equation modeling

## Abstract

**Background:**

Little is known about associations of psychological and mental well-being with cognitive and socioemotional factors in low and middle-income countries, particularly among vulnerable populations born in adverse environments that may restrict developmental potential. This study aimed to examine the cognitive and socioemotional correlates of psychological well-being and mental health in a cohort of Guatemalan adults born in contexts of poverty and malnutrition.

**Methods:**

From Dec 2017 to Apr 2019, data were collected from 704 women and 564 men ages 40–57 years living in four rural villages in eastern Guatemala and Guatemala City. We measured latent domains of psychological well-being, spirituality and religion, emotional support, and executive function using Confirmatory Factor Analysis (CFA). Under a Structural Equation Modeling framework, we examined intercorrelations among latent domains and observed measures of intelligence and mental health.

**Results:**

CFA supported the construct validity of factor structures in this population. Correlations of psychological well-being with spirituality and religion were moderate in women (*r* = 0.68, *p* < 0.001) and men (*r* = 0.70, *p* < 0.001). Executive function was weakly correlated with psychological well-being in men (*r* = 0.23, *p* < 0.001) and showed no association in women. Correlations of psychological well-being with emotional support and IQ were weak in women (*r* = 0.34, and *r* = 0.15, respectively; *p* < 0.001 for both) and men (*r* = 0.35, and *r* = 0.25, respectively; *p* < 0.001 for both). Mental health and IQ were weakly correlated in men (*r* = 0.09, *p* < 0.05) and showed no association in women. Mental health showed weak correlations with emotional support (*r* = 0.18, *p* < 0.001 in women; *r* = 0.09, *p* < 0.05 in men), psychological well-being (*r* = 0.32 and *r* = 0.35, in women and men respectively; *p* < 0.001 for both) and showed no association with executive function in both sexes.

**Conclusions:**

Of all examined factors, spirituality and religion made the greatest contribution to psychological well-being. These findings support the notion that in populations experiencing difficult circumstances, religion can perhaps make a greater contribution to well-being and aid coping. More research is needed to examine mediators of this association.

**Supplementary Information:**

The online version contains supplementary material available at 10.1186/s40359-021-00654-y.

## Background

Psychological well-being is a multidimensional construct that refers to optimal psychological functioning and experience [1]. The use of subjective well-being as the overall measure of prosperity has gained prominence over the last few years, moving away from traditional macro-economic indicators like GDP [2]. Much evidence suggests that some of the skills that could contribute to achieving psychological well-being rely on higher-order cognitive processes of general intelligence [3–5] and executive function [6–8]. General intelligence, usually measured using intelligence quotient (IQ) tests, is the ability to acquire knowledge and use it in novel ways [9]. Executive function refers to the cognitive process responsible for controlling and regulating thoughts, emotions, and behaviors in pursuit of personal goals [10].

Much of what is known about intelligence and executive function derives from studies conducted in Western, Educated, Industrialized, Rich and Democratic (WEIRD) countries [11]. This is a limitation to the generalizability of cognitive models because it remains unclear whether they apply to non-Western contexts. In addition, the limitations of applying tests of cognitive ability from one ethnic group to another without appropriate standardization are well-recognized [12, 13].

Although most research on intelligence and executive function has been conducted in high-income countries, growing evidence points to similar findings in low- and middle-income countries (LMICs), including links between IQ and executive function with psychological, social, and mental health outcomes. These studies show that higher IQ predicts better income [3, 5] education attainment, reduced criminal and delinquent involvement [5], and higher life satisfaction [4]. Similarly, higher executive function has been associated with academic achievement [6], higher income, lower substance abuse, and criminal offending outcomes [7]. Executive function has also been linked to mental health outcomes [8], with studies suggesting that it serves as an important cognitive resource, involved in the ability to cope with stressful events and regulate mood and thoughts [14].

A substantial body of research has examined the influence of coping resources (i.e., social support, religion, and spirituality) on promoting psychological well-being. In LMICs, positive links of social support with subjective well-being and mental health outcomes have been reported [15–18]. Similarly, studies have found that people with strong spiritual or religious faith report higher levels of happiness, life satisfaction [19], fewer symptoms of depression [20, 21], and substance abuse [21], and have higher levels of social support [22].

While much about the association of subjective well-being and mental health with cognitive and socioemotional factors has been investigated, most studies have been conducted in developed countries and populations experiencing psychiatric and medical conditions or among the elderly. Very little research has been conducted among populations born in contexts of poverty and malnutrition that may restrict developmental potential. It is well established that childhood adversities leave long-lasting imprints on the neural mechanisms of cognition and emotion [23, 24]. Thus, it is important to examine the factors that could influence psychological well-being and mental health in these populations. This information could provide valuable data to inform the future development of targeted intervention programs.

The objective of this study is to examine the cognitive and socioemotional correlates of psychological well-being and mental health in a cohort of Guatemalan adults who were born in contexts of poverty and malnutrition. This study focuses on a small subset of factors that have been shown to influence psychological wellbeing. Except for emotional support, this study does not address socioecological aspects.

## Methods

### Study population and setting

The population in our study had participated in a community-randomized food supplementation trial in early childhood. The intervention was implemented between 1969 and 1977 by the Institute of Nutrition of Central America and Panama (INCAP) in 4 rural communities in eastern Guatemala (n = 2392). At the time of the intervention, child undernutrition and infectious diseases were endemic in the study villages, and most adults were illiterate [25]. The nutritional trial was designed to assess the impact of improved nutrition on child growth and cognitive development. The communities where the study was conducted were predominantly ladino (mixed Spanish and indigenous descent) with a very low Mayan population density. Participants in the study were also Ladino, as a result all participants were Spanish speaking.

Complete details of the original trial and subsequent follow-up studies are published elsewhere [26, 27].

### Training and data collection

This paper utilizes data collected from Dec 2017 to Apr 2019 in 1268 participants, with a mean age of 47 years. In the 2017–19 follow-up, out of the 1643 cohort members presumed alive and living in Guatemala (68.7% of the original cohort), 261 declined or were physically unable to participate, and 114 could not be contacted after multiple attempts. Apart from sex, characteristics of participants who were lost to follow-up were similar to those who participated in the 2017–19 study (Additional file 1: Table 1).

All survey instruments (details below) were tested before the commencement of the study. Participants were interviewed in a research facility established in a rented building in each village or at INCAP headquarters in Guatemala City. Survey instruments were administered by trained enumerators. Details on training, adaptation, and administration of the cognitive tests are published elsewhere [28].

The Institutional Review Boards of Emory University (Atlanta, GA) and INCAP (Guatemala City, Guatemala) gave ethical approval for this study. All methods were carried out in accordance with relevant guidelines and regulations and all participants gave written informed consent.

### Measurements

#### Psychological well-being

We adopted a model that captures both hedonic and eudaimonic aspects of psychological well-being. The hedonic approach defines well-being in terms of pleasure attainment and pain avoidance (e.g., happiness; life satisfaction), and the eudaimonic approach defines well-being in terms of psychosocial functioning (e.g., meaning and purpose; self-efficacy) [1, 29, 30].

We measured happiness using the Lyubomirsky Scale of Global Subjective Happiness [31]. Participants were asked to rate four items on a 5-point Likert scale ranging from 1 (*Very unhappy* or *not at all*) to 5 (*Very happy* or *a great deal*). A total score was calculated by computing the mean of the four items, with higher scores signifying greater happiness [31].

We assessed life satisfaction using the NIH Toolbox General Life Satisfaction Survey consisting of five items assessing global feelings and attitudes about one's life [32]. Participants rated these items on a 5-point Likert scale ranging from 1 (*Strongly disagree*) to 5 (*Strongly agree*). Final scores were computed as the sum of item scores, with higher scores signifying higher life satisfaction.

Meaning and purpose in life was measured using the NIH Toolbox Meaning and Purpose Survey. Participants rated nine items on a 5-point Likert scale ranging from 1 (*Strongly disagree*) to 5 (*Strongly agree*) [32]. Final scores were computed as the sum of item scores, with higher scores signifying greater meaning and purpose.

Self-efficacy is defined as a person's belief in his/her capacity to manage, function, and have control over meaningful events [33]. We assessed self-efficacy using the NIH Toolbox Self-Efficacy Survey [34]. Participants rated ten items on a 5-point Likert scale ranging from 1 (*Never*) to 5 (*Very often*). Final scores were computed as the sum of item scores, with higher scores signifying greater self-efficacy.

#### Socio-emotional measures

##### Emotional support

Emotional support is one component of social support that refers to the experience of being cared about, valued, and loved by people in one's social network [35]. We assessed emotional support using the fixed 8-item form of the NIH Toolbox Emotional Support Survey [36]. Each item administered has a 5-point Likert scale. Total scores were the sum of all items, with higher scores signifying higher emotional support.

##### Spirituality and religion

While the terms spirituality and religion are used interchangeably, they have different meanings. Religion functions in the context of an organized institution that places spirituality under a specific set of beliefs, values, and practices. On the other hand, spirituality is a subjective experience that involves a sense of connection and transcendence with a greater force [37]. Thus, religion can be considered a specific form of spirituality, while spirituality is a broader concept.

We assessed spirituality and religion using the faith and hope facets of the World Health Organization Quality of Life Spirituality, Religiousness, and Personal Beliefs WHOQOL-SRPB questionnaire [38]. The WHOQOL-SRPB questionnaire does not tie spirituality to religion, and questions are phrased in ways that apply to individuals with a wide range of religious and non-religious beliefs. Each facet included 4-items on a 5-point Likert scale and was scored through summative scaling, with each item contributing equally to the facet score. Mean scores were then calculated, where higher values reflect higher levels of spirituality and religion [38].

#### Mental health

We used the WHO Self-Reporting Questionnaire (SRQ-20) to assess mental health. The SRQ-20 is a screening tool for mental disorders specifically designed for developing countries, consisting of 20 *yes/no* questions, with a maximum score of 20 [39]. Higher values are indicative of worse symptomology. For consistency with the other measures collected, we reverse-scored the items so that higher mean values are indicative of greater mental health.

#### Cognitive measures

##### Non-verbal fluid intelligence (IQ)

We assessed non-verbal fluid intelligence using the Raven’s Progressive Matrices Test [40]. The test consists of a series of increasingly complex patterns, for each of which there is a piece missing. Participants were asked to select which piece completes the pattern from several options. Three of the five scales (A, B, and C, with 12 questions each) were administered since previous administrations of the instrument in this population showed that only few participants could progress beyond the third scale. Scores were computed as the sum of correct responses, for a maximum total score of 36. Since language is not required for its administration, the Raven’s Progressive Matrices Test is typically viewed as a relatively culture-fair test [41].

##### Executive function

We measured executive function as a latent construct that includes working memory, inhibitory control, and cognitive flexibility as building blocks [42, 43]. We administered computerized Spanish-language tests of the National Institutes of Health (NIH) Toolbox cognition battery. To assess working memory capacity, we used the List Sorting Working Memory Test [44]. The task requires participants to remember information that is visually and auditorily presented with illustrated pictures (either foods, animals, or both) in size order from the smallest to the largest. Item substitutions were made to the List Sorting Working Memory test to increase cultural appropriateness. Specifically, pumpkin was substituted with papaya, cherry with nispero (loquat), and blueberry with nance (a small tropical fruit). These adaptations were approved and implemented by the NIH Toolbox development team. Final scores were computed as the sum of correct responses for a maximum score of 26. Inhibitory control was assessed using the Flanker Inhibitory Control and Attention Test [45]. The task requires participants to indicate the left–right orientation of a centrally presented stimulus while inhibiting potentially irrelevant information from the flanking stimuli [46]. We used the Dimensional Change Card Sort (DCCS) Test to assess cognitive flexibility. Participants were asked to switch between matching pictures by color and matching pictures by shape [45]. For Flanker Inhibitory Control and Attention and DCCS Tests, we used the NIH toolbox computed scores, which uses a two-vector algorithm that combines accuracy and reaction time [47]. Higher values indicate greater cognitive flexibility and inhibitory control. Computed scores range from 0 to 10, and a score between 0 and 5 indicates that the participant did not score high enough in accuracy (80% correct or less).

### Statistical analysis

In all scales with missing items (< 1%), we applied a two-way imputation approach [48]. We used Structural Equation Modelling (SEM) techniques to examine the factor-loadings patterns of studied domains and to assess their intercorrelations. Under the SEM framework, we used first-order Confirmatory Factor Analysis (CFA) to investigate whether the established dimensionality and factor-loadings patterns for executive function and scales assessing happiness, life satisfaction, meaning and purpose, self-efficacy, emotional support, faith and hope fitted our sample population. For second-order factor structures (i.e., psychological well-being and spirituality and religion), we used hierarchical CFA to determine the degree to which factors loaded on their underlying sub-constructs.

**Fig. 1:**
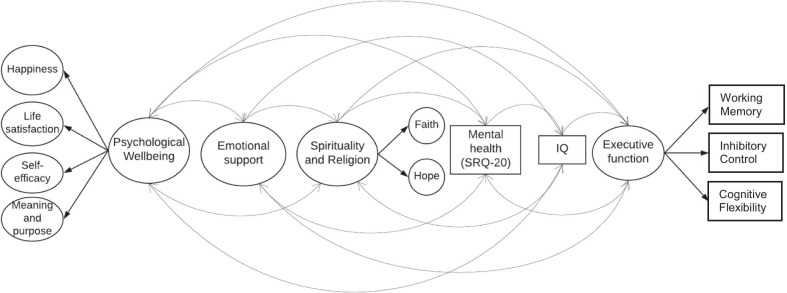
Hypothesized model examining cognitive and socioemotional correlates of psychological well-being and mental health in a cohort of Guatemalan adults

Our final model included the individual Likert-item responses from the scales assessing happiness, life satisfaction, meaning and purpose, and self-efficacy as latent sub-domains of “psychological well-being." Likert-item responses from the NIH Emotional Support Scale were used to model emotional support. Likert-item scale responses from the faith and hope facets of the WHOQOL-SRPB scale were modeled as latent sub-domains of “spirituality and religion." Mental health and IQ were modeled as observed variables using computed scores. List Sorting Working Memory, Flanker Inhibitory Control and Attention, and DCCS Tests scores (cognitive flexibility) were modeled as latent "executive function." (Fig. 1).

Additionally, for comparison purposes, we tested a model in which the hope and faith facets were modeled together with happiness, life satisfaction, meaning and purpose, and self-efficacy as additional subdomains of psychological well-being.

We assessed model fit using the root mean square error of approximation (RMSEA), Comparative Fit Index (CFI), and Tucker–Lewis index (TLI). A good model fit is indicated by RMSEA < 0.08, CFI > 0.90 and TLI > 0.95.

We interpreted correlation coefficients > 0.70 as strong, between 0.70 and 0.40 as moderate, and < 0.40 as weak.

Our analysis accounted for clustering of subjects within family, and models were sex stratified. All analysis was conducted using MPLUS 8.0 using the Weighted Least Square Mean and Variance (WLSMV) estimator for categorical and ordinal responses using pairwise deletion of missing values [49].

## Results

Study participants (704 women; 564 men) had a mean age of 47 years. Table 1 shows means and SD of the scores of completed tests. Models in women and men showed adequate fit (RMSEA = 0.04; CFI = 0.95, TLI = 0.95, and RMSEA = 0.04, CFI = 0.95, TLI = 0.94, respectively).

**Table 1:** Demographic, cognitive, and socioemotional characteristics of the study population, by sex^a^

	n	Women	n	Men	*p*-value
Age, y	704	47.5 ± 4.3	564	47.4 ± 4.1	0.61
Area of residence, % rural	704	71.6	564	74.6	0.23
Psychological well-being					
Lyubomirsky Happiness (out of 5)	700	4.0 ± 1.0	553	4.1 ± 0.9	0.25
NIH Life Satisfaction (out of 25)	699	18.5 ± 3.5	553	18.9 ± 3.4	0.07
NIH Meaning and Purpose (out of 45)	698	36.2 ± 4.1	552	37.2 ± 4.0	< 0.01
NIH Self-efficacy (out of 40)	698	30.9 ± 7.1	552	31.4 ± 6.4	0.25
Socio-emotional scores					
NIH Emotional support (out of 40)	703	31.0 ± 9.3	559	32.5 ± 7.7	< 0.01
WHOQOL-SRPB Hope facet (out of 5)	698	3.2 ± 0.8	552	3.3 ± 0.7	< 0.05
WHOQOL-SRPB Faith facet (out of 5)	698	3.9 ± 0.7	552	3.9 ± 0.7	0.79
Mental health					
SRQ-20 (out of 20)^b^	700	15.2 ± 4.0	558	17.7 ± 2.9	< 0.01
Cognitive tests scores					
Intelligence					
Raven's Progressive Matrices (# correct out of 36)	686	15.3 ± 4.9	537	17.8 ± 6.0	< 0.01
Executive function					
List Sorting Working Memory (# correct out of 26)	670	11.3 ± 3.8	541	12.7 ± 3.9	< 0.01
Flanker Inhibitory Control and Attention (NIH score)^c^	671	5.4 ± 1.1	542	5.8 ± 1.2	< 0.01
Cognitive flexibility—DCCS (NIH score)^c^	677	5.1 ± 1.9	546	5.4 ± 1.9	< 0.01

*NIH* National Institutes of Health, *DCCS* Dimensional Change Card Sort, *WHOQoL SRPB* World Health Organization Quality of Life Spirituality, Religiosity, and Personal Beliefs, *SRQ-20* Self-Reported Questionnaire-20

^a^Values are means ± SD or percentages

^b^For interpretation purposes items were reverse scored so that higher mean values are indicative of greater mental health

^c^Computed scores range from 0 to 10, but if the score is between 0 and 5, it indicates that the participant did not score high enough in accuracy (80% correct or less)

Models combining the hope and faith facets with the psychological well-being components indicated a small decrease in goodness-of-fit indices (RMSEA = 0.05, CFI = 0.94, TLI = 0.93 in women, and RMSEA = 0.04, CFI = 0.94 and TLI = 0.93 in men). Thus, we decided to keep the model that differentiates spirituality and religion from psychological well-being.

First-order factor loadings for scales assessing happiness, life satisfaction, meaning and purpose, self-efficacy, emotional support, hope, and faith are presented in Additional file 1: Table 2. Second-order CFA showed that the theorized subcomponents for psychological well-being, and spirituality and religion were highly loaded into their underlying constructs. We also found computed scores for List Sorting Working Memory, Flanker Inhibitory Control and Attention, and DCCS tests loaded onto the executive function latent construct (Table 2).

**Table 2:** Factor loadings for psychological well-being, spirituality, and religion, and executive function latent constructs^a^

	Women	Men
Psychological well-being		
Lyubomirsky happiness	0.73	0.69
NIH life satisfaction	0.86	0.86
NIH meaning and purpose	0.83	0.83
NIH self-efficacy	0.55	0.53
Spirituality and religion^b^		
Faith	0.79	0.73
Hope	0.91	0.88
Executive function		
List sorting working memory test	0.60	0.66
Flanker Inhibitory control and attention test	0.59	0.69
Cognitive flexibility—DCCS^c^ test	0.82	0.70

^a^All factor loadings are statistically significant (*p* < 0.01)

^b^Measured using the hope and faith facets of the World Health Organization Quality of Life Spirituality, Religiosity and Personal Beliefs (WHOQoL SRPB)

^c^DCCS Dimensional Change Card Sort (DCCS)

Intercorrelation matrices between latent domains and observed variables in women and men are presented in Tables 3 and 4, respectively. In women, psychological well-being was moderately associated with spirituality and religion (*r* = 0.68, *p* < 0.001), weakly correlated with emotional support (*r* = 0.34, *p* < 0.001), mental health (*r* = 0.32, *p* < 0.001) and IQ (*r* = 0.15, *p* < 0.001), and showed no association with executive function. Mental health was weakly correlated with emotional support (*r* = 0.18, *p* < 0.001), spirituality, and religion (*r* = 0.16, *p* < 0.001), and showed no association with IQ and executive function. We also found moderate correlations between executive function and IQ (*r* = 0.63, *p* < 0.001) (Table 3).

**Table 3:** Correlation matrix of cognitive and socioemotional domains among adult women in Guatemala (n = 704)

	Psychological well-being	Emotional support	Spirituality and religion	Mental health	IQ	Executive function
Psychological well-being	–					
Emotional support	0.34**	–				
Spirituality and religion	0.68**	0.19**	–			
Mental health	0.32**	0.18**	0.16**	–		
IQ	0.15**	0.09*	0.27**	0.08	–	
Executive function	0.08	0.15**	0.38**	0.08	0.63**	–

^*^p < 0.05; **p < 0.001

**Table 4:** Correlation matrix of cognitive and socioemotional domains among adult men in Guatemala (n = 564)

	Psychological well-being	Emotional support	Spirituality and religion	Mental health	IQ	Executive function
Psychological well-being	–					
Emotional support	0.35**	–				
Spirituality and religion	0.70**	0.32**	–			
Mental health	0.35**	0.09*	0.12*	–		
IQ	0.25**	0.11*	0.32**	0.09*	–	
Executive function	0.23**	0.22**	0.43**	0.08	0.70**	–

^*^p < 0.05; **p < 0.001

The correlation matrix in men showed similar results. We observed a moderate correlation between psychological well-being and spirituality and religion (*r* = 0.70, *p* < 0.001). Psychological well-being was weakly correlated with emotional support (*r* = 0.35, *p* < 0.001), mental health (*r* = 0.35, *p* < 0.001), IQ (*r* = 0.25, *p* < 0.001) and executive function (*r* = 0.23, *p* < 0.001). Mental health was weakly associated with emotional support (*r* = 0.09, *p* < 0.05), spirituality and religion (*r* = 0.12, *p* < 0.05), and IQ ( *r* = 0.09, *p* < 0.05), and showed no association with executive function. We also found moderate correlations between executive function and IQ (*r* = 0.70, *p* < 0.001) (Table 4).

## Discussion

We investigated associations of psychological well-being and mental health with executive function, IQ, spirituality and religion, and emotional support in a population of Guatemalan adults born in contexts of poverty and malnutrition. Our results derived from CFA support the use of the applied measures in this context. Our findings demonstrate the construct validity of first order (i.e., happiness, life satisfaction, meaning and purpose, self-efficacy, emotional support, faith, hope and executive function) and second order (i.e., psychological well-being and spirituality and religion) factor structures. In both sexes, spirituality and religion was moderately correlated with psychological well-being and weakly correlated with mental health. Much debate has revolved around whether there is a meaningful differentiation between spirituality and religion from psychological well-being components. In agreement with previous studies [50], our findings support differentiation between these two constructs.

The positive association of psychological well-being with spirituality and religion is well documented [51–53]. Our study findings show positive correlations of spirituality and religion with subjective well-being (r = 0.68 in women and r = 0.70 in men), that are in line with previous research conducted in non-Western contexts, indicating small but consistent positive associations between religiosity and psychological well-being [19, 53], even after controlling for difficult life circumstances [53]. Using data from 153 nations, Diener and collaborators found that in religious societies experiencing difficult life circumstances (e.g., poverty, low education, malnutrition, low life expectancy, etc.), religious individuals had greater levels of subjective well-being than non-religious individuals, and this association was mediated by social support, respect and purpose in life [53]. It is possible that organized religion provides supportive social structures that can, to some extent, diminish the harmful effects of difficult life circumstances. Furthermore, the authors found that difficult individual circumstances were associated with religiosity at the individual level (r = 0.29) and country level (r = 0.65), suggesting that difficult life circumstances could lead to greater religiosity [53].

The mechanism by which spirituality and religion could influence psychological well-being has been suggested to involve psychosocial factors such as providing a sense of identity and social support and promoting an active and socially engaged lifestyle [54]. Our study found weak associations of emotional support (our measure of social support) with psychological well-being, and spirituality and religion, in both sexes. However, our social support measure was limited to emotional aspects and did not include components of instrumental support or social networks, which could be underestimating the associations. The religious landscape in Guatemala may provide additional insights into the observed associations. Pentecostal congregations rose in popularity in Guatemala during the late 1970s, turning it into one of the most Protestant countries in Latin America [55]. This is relevant because pentecostal churches are very supportive of their adherents, providing them with various social services. The extent to which social support mediates the association of spirituality and religion with psychological well-being in this population remains to be investigated.

We measured three core executive functions (working memory, inhibitory control, and cognitive flexibility) that facilitate higher-order executive function: problem-solving, reasoning, and planning. It is well established that the cognitive processes involved in executive function are critical to mental health and psychological well-being. Research has shown that people show better executive function capacities when they feel happy, socially supported, and healthy [56]. Conversely, deficits in executive function have been associated with depression, and various psychopathologies [57]. Our findings suggest that in this population of Guatemalan adults, executive function had little if any association with psychological well-being and mental health constructs. The mechanisms through which greater executive function positively influence various aspects of well-being (e.g., good relationships, health, and academic achievement) have been shown to involve the ability to inhibit automatic responses (i.e., self-control) and delay of gratification [58]. These proposed mechanisms have been derived from studies conducted on children and adolescents in high-income countries [59–62]. More cross-cultural research is needed to understand the underlying mechanisms influencing better outcomes among those with higher executive function.

We also found that executive function was moderately correlated with spirituality and religion. These findings are in line with previous studies documenting associations of spiritual and religious involvement with better inhibitory control and lower cognitive decline [63]. Proposed mechanisms include the stimulation of higher cortical functions related to abstract thinking [64] and promoting a stimulating and socially engaged lifestyle that may help prevent cognitive decline [63].

Our study also showed that executive function was moderately and positively correlated with general intelligence measures (IQ). These findings are consistent with previous studies indicating that performance on executive function tests, particularly on tasks assessing working memory capacity, is associated with measures of intelligence [65, 66].

There are important considerations to be made given the well-known debate around cultural bias in cross-cultural assessment [67]. Most of the measures used in our study derived from the Spanish-language version of the NIH Toolbox. These measures were evaluated for cultural appropriateness with Hispanics/Latinos living in the United States whose primary language was Spanish [68]. The assessment of the Spanish-language version of the NIH Toolbox showed good psychometric properties and supports its use to measure cognitive and behavioral functioning among Spanish-speaking individuals in the United States [69]. We investigated the construct validity of these measures in our study population using CFA. Our results supported their factor structure, but it should be acknowledged that this method of validation is limited for cross-cultural settings. Ideally, a cross-cultural validation should be implemented using quantitative and qualitative methods, and measures compared against a gold standard [67]. Our study is also limited by the data's cross-sectional nature, which does not allow for directionality or causality to be inferred. Another limitation is the limited generalizability of our findings, which may only apply to populations with similar characteristics to those of our study sample.

Our study also has strengths. We applied cognitive and socio-emotional measures with good psychometric properties in a large sample of men and women living in rural areas of Guatemala or Guatemala City. Moreover, structural equation modeling techniques allow examining interrelationships among factors and observed variables while accounting for measurement error.

## Conclusions

Our findings contribute to the understanding of factors that could help strengthen psychological well-being in populations born in contexts of poverty and malnutrition in LMICs. Our findings suggest that spirituality and religion may help people cope with difficult life circumstances. Future research should examine mediators of this association and use longitudinal designs to determine the directionality of the religiosity and subjective well-being relationship.

## Supplementary Information

**Additional file 1.** Supplementary material.

## Data Availability

The datasets used and/or analyzed during the current study are available from the corresponding author on reasonable request.

## Abbreviations

CFA: Confirmatory Factor Analysis
CFI: Comparative Fit Index
DCCS: Dimensional Change Card Sort
INCAP: Institute of Nutrition of Central America and Panama
IQ: Intelligence quotient
LMICs: Low-and middle-income countries
NIH: National Institutes of Health
RMSEA: Root mean square error of approximation
SEM: Structural Equation Modelling
SRQ-20: WHO Self-Reporting Questionnaire
TLI: Tucker–Lewis index
WHOQOL-SRPB: World Health Organization Quality of Life Spirituality, Religiousness and Personal Beliefs questionnaire
WLSMV: Weighted Least Square Mean and Variance
