# Supplementary material for: Cognitive and socio-emotional correlates of psychological well-being and mental health in Guatemalan adults
Source: BMC Psychol. 2021 Sep 23;9:148. doi: 10.1186/s40359-021-00654-y (PMC8459534; doi:10.1186/s40359-021-00654-y)
Supplement: Supplementary file 1 — Additional file 1. Supplementary material. [file 40359_2021_654_MOESM1_ESM.docx]

**Cognitive and socio-emotional correlates of psychological well-being and mental health in Guatemalan adults**

María J. Ramírez-Luzuriaga ^1^

Laura Ochaeta ^2^

Manuel Ramírez-Zea ^2^

Ann DiGirolamo ^3^

Rachel Waford ^1,4^

Charlotte Wray ^5^

Reynaldo Martorell ^1,4^

Aryeh D. Stein ^1,4^

^1^ Nutrition and Health Science Program, Laney Graduate School. Emory University. Atlanta,

GA, USA.

^2^ INCAP Research Center for the Prevention of Chronic Diseases (CIIPEC), Institute of

Nutrition of Central America and Panama, Guatemala City, Guatemala.

^3^ Georgia Health Policy Center, Georgia State University. Atlanta, GA, USA.

^4^ Hubert Department of Global Health, Rollins School of Public Health Emory University.

Atlanta, GA, USA

^5^ Department of Psychiatry, Medical Sciences Division, University of Oxford, Oxford, UK.

Correspondence concerning this article should be addressed to Aryeh. D. Stein, Room 7007, 1518 Clifton Rd NE, Atlanta GA 30322, E-mail: [aryeh.stein@emory.edu](mailto:aryeh.stein@emory.edu)

**Supplemental Table 1** Characteristics of participants in the trial lost to follow up ^1^

|  | **Death** | | **International Migration** | | **Did not otherwise participate in 2017-19 study wave** | | **Participated in 2017-19 study wave** | | **Total** | |
| --- | --- | --- | --- | --- | --- | --- | --- | --- | --- | --- |
|  | **n** |  | **n** |  | **n** |  | **n** |  | **n** |  |
| Female, % | 385 | 41.6 | 252 | 34.9 | 487 | 43.1 | 1268 | 55.5 | 2392 | 48.6 |
| Year of birth | 385 | 1971  (1968, 1974) | 252 | 1971  (1967, 1974) | 487 | 1970  (1967, 1973) | 1268 | 1970  (1967, 1974) | 2392 | 1971  (1967, 1974) |
| Childhood household SES, SD | 385 | -0.01  (-0.61, 0.52) | 252 | 0.08  (-0.48, 0.73) | 487 | -0.01  (-0.58, 0.52) | 1268 | -0.09  (-0.69, 0.39) | 2392 | -0.03  (-0.61, 0.52) |
| Maternal age, y | 378 | 26  (21, 33) | 248 | 25  (21, 32) | 459 | 26  (21, 32) | 1259 | 26  (21, 32) | 2344 | 26  (21, 32) |
| Maternal height, cm | 295 | 148  (144, 152) | 187 | 149  (146, 152) | 318 | 149  (146, 152) | 997 | 148  (145, 152) | 1799 | 148  (145, 152) |
| Maternal schooling, y | 336 | 0.0  (0.0, 2.0) | 228 | 1.0  (0.0, 2.0) | 382 | 0.0  (0.0, 2.0) | 1223 | 1.0  (0.0, 2.0) | 2169 | 1.0  (0.0, 2.0) |

^1^Values presented are medians (25^th^, 75^th^ percentiles) or percents

**Supplemental Table 2** First-order factor loadings for socio-emotional scales, by gender ^1^

|  | **Women** | **Men** |
| --- | --- | --- |
| Happiness ^2^ |  |  |
| 1. How happy would you rate yourself? | 0.86 | 0.86 |
| 1. How happy would you rate yourself compared to most of your peers? | 0.89 | 0.77 |
| 1. Some people are generally very happy. They enjoy life regardless of what is going on, getting the most out of everything. To what extent does this characterization describe you. | 0.72 | 0.69 |
| 1. Some people are generally not very happy. Although they are not depressed, they never seem as happy as they might be. To what extent does this characterization describe you. | 0.40 | 0.47 |
| Life Satisfaction ^3^ |  |  |
| 1. My life is going well | 0.79 | 0.82 |
| 1. My life is just right | 0.79 | 0.76 |
| 1. I wish I had a different kind of life | 0.39 | 0.28 |
| 1. I have a good life | 0.83 | 0.87 |
| 1. I have what I want in life | 0.79 | 0.77 |
| Meaning and Purpose ^4^ |  |  |
| 1. I understand my life’s meaning | 0.82 | 0.83 |
| 1. My life has a clear sense of purpose | 0.78 | 0.78 |
| 1. I have a good sense of what makes my life meaningful | 0.76 | 0.80 |
| 1. I have discovered a satisfying life purpose | 0.81 | 0.80 |
| 1. My life has no clear purpose | 0.28 | 0.41 |
| 1. I generally feel that what I do in my life is valuable and worthwhile | 0.81 | 0.86 |
| 1. I feel grateful for each day | 0.71 | 0.77 |
| 1. My daily life is full of things that are interesting to me | 0.83 | 0.80 |
| 1. There is not enough purpose in my life | 0.27 | 0.36 |
| Self-efficacy ^5^ |  |  |
| 1. I can manage to solve difficult problems if I try hard enough | 0.71 | 0.72 |
| 1. If someone opposes me, I can find the means and ways to get what I want | 0.70 | 0.59 |
| 1. It is easy for me to stick to my aims and accomplish my goals | 0.75 | 0.70 |
| 1. I am confident that I could deal efficiently with unexpected events | 0.78 | 0.71 |
| 1. Thanks to my talents and skills, I know how to handle unexpected situations | 0.82 | 0.76 |
| 1. I can solve most problems if I try hard enough | 0.76 | 0.75 |
| 1. I stay calm when facing difficulties because I can handle them | 0.75 | 0.74 |
| 1. When I have a problem, I can find several ways to solve it | 0.78 | 0.75 |
| 1. If I am in trouble, I can think of a solution | 0.77 | 0.71 |
| 1. I can handle whatever comes my way | 0.73 | 0.66 |
| Emotional Support ^6^ |  |  |
| 1. I have someone who understands my problems | 0.76 | 0.77 |
| 1. I have someone who will listen to me when I need to talk | 0.84 | 0.80 |
| 1. I feel there are people I can talk to if I am upset | 0.89 | 0.79 |
| 1. I have someone to talk with when I have a bad day | 0.87 | 0.79 |
| 1. I have someone I trust to talk with about my problems | 0.87 | 0.87 |
| 1. I have someone I trust to talk with about my feelings | 0.87 | 0.82 |
| 1. I can get helpful advice from others when dealing with a problem | 0.83 | 0.76 |
| 1. I have someone to turn to for suggestions about how to deal with a problem | 0.76 | 0.70 |
| Hope ^7^ |  |  |
| 1. How hopeful do you feel? | 0.78 | 0.72 |
| 1. To what extent are you hopeful about your life? | 0.78 | 0.83 |
| 1. To what extent does being optimistic improve your quality of life? | 0.84 | 0.73 |
| 1. How able are you to remain optimistic in times of uncertainty? | 0.77 | 0.72 |
| Faith ^8^ |  |  |
| 1. To what extent does faith contribute to your well-being? | 0.88 | 0.81 |
| 1. To what extent does faith give you comfort in daily life? | 0.89 | 0.91 |
| 1. To what extent does faith contribute to your well-being? | 0.90 | 0.88 |
| 1. To what extent does faith give you strength in daily life? | 0.79 | 0.80 |

^1^All factor loadings are statistically significant, p<0.01; ^2^ Lyubomirsky Subjective Happiness Scale;

^3^ National Institutes of Health (NIH) Life Satisfaction Survey; ^4^ NIH Meaning and Purpose Survey;

^5^ NIH Self-efficacy Survey; ^6^ NIH Emotional Support; ^7^ Hope facets of the World Health Organization Quality of Life Spirituality, Religiosity and Personal Beliefs (WHOQoL SRPB); ^8^ Faith facet of the WHOQoL SRPB.
